# Alcohol: A Nutrient with Multiple Salutary Effects

**DOI:** 10.3390/nu7031992

**Published:** 2015-03-18

**Authors:** Henry J. Pownall, Corina Rosales, Baiba K. Gillard, Antonio M. Gotto

**Affiliations:** 1 Department of Cardiology, Division of Atherosclerosis, Houston Methodist Research Institute, Fondren F8-047, 6670 Bertner St., Houston, TX 77030, USA; E-Mails: crosales@houstonmethodist.org (C.R.); bgillard@houstonmethodist.org (B.K.G.); amg2004@med.cornell.edu (A.M.G.); 2 Department of Medicine, Weill Cornell Medical College, 1305 York Ave. Y-805, New York, NY 10021, USA

**Keywords:** Alcohol, acetate, insulin resistance, HDL, atherosclerosis

## Abstract

Numerous studies have shown that cardiovascular disease is lower among alcohol consumers than among nonconsumers. Many of the metabolic effects of alcohol are mediated by its terminal metabolite, acetate, which has reported insulinemic properties. There have been few rational metabolic targets that underly its cardioprotective effects until it was reported that acetate, the terminal product of alcohol metabolism, is the ligand for G-protein coupled receptor 43 (GPCR43), which is highly expressed in adipose tissue. Here, we recast much of some of the major lipid and lipoprotein effects of alcohol in the context of this newly discovered G-protein and develop a mechanistic model connecting the interaction of acetate with adipose tissue-GPCR43 with these effects. According to our model, ingestions of acetate could replace alcohol as a means of improving plasma lipid risk factors, improving glucose disposal, and reducing cardiovascular disease. Future studies should include biochemical, cell, animal, and human tests of acetate on energy metabolism.

## 1. Alcohol in Health and Disease

Wine, beer, and spirits have been a component of the diet in many cultures over the ages. In moderation, alcoholic beverages have been used to enhance social interactions and the enjoyment of food. Thus, alcoholic beverages have for the most part been used for pleasure and relaxation. Alcohol has one well-known salutary effect; people who consume alcohol in moderation, two drinks/day for men and one drink/day for women, live longer and experience less cardiovascular disease (CVD); with higher alcohol intake life expectancy decreases, an effect that is due not to CVD but rather to well-known alcohol-promoted conditions, cirrhosis, and cancers of the mouth, esophagus, pharynx, larynx, and liver; breast cancer in women; injuries and other external causes in men [1]. These data and many other observational studies would suggest that moderate alcohol consumption is healthful. However, many questions remain. What are the mechanisms by which alcohol reduces the CVD rates? Are there other healthful alternatives to alcohol consumption that could provide the positive CVD effects of alcohol without the occurrence of other diseases, including psychosocial effects that occur at high rates of consumption?

## 2. Alcohol and Plasma Lipoproteins

### 2.1. Alcohol and Endogenous Triglyceride Metabolism

Chronic addition of ethanol to a normal diet (~90 g over three feedings daily for seven days) produces a mild hypertriglyceridemic effect, which is more profound among hypertriglyceridemic patients [2]. Some clues to the underlying cause of lipemia may be found in studies of acute ethanol intake [3]. Intravenous alcohol intake profoundly reduces plasma free fatty acids and glycerol, suggestive of inhibited lipolysis; plasma glucose levels are not affected and the plasma fatty acid concentrations returned to baseline levels within one hour. This effect, which is well-known among diabetic patients, is due to increased insulin secretion [4]. Coadministration of alcohol and epinephrine reduces plasma free fatty acid levels, suggesting inhibited adipose tissue lipolysis. Interestingly, infusion of the terminal ethanol metabolite, acetate, produced effects similar to the alcohol infusions, suggesting that the active agent in the alcohol-mediated reduction of plasma free fatty acids is acetate, an effect supported by *in vitro* studies. Comparison of the effects of alcohol with those of its two serially formed products showed that acetate but not alcohol nor acetaldehyde inhibited lipolysis in isolated rat adipocytes [5].

### 2.2. Effects of Alcohol on Postprandial Lipemia

Immediately following a fat-containing meal, plasma triglycerides rise over a period of hours, peaking at 4 hours and returning to baseline at 8 hours. This effect is more profound for CVD patients *vs.* controls [6], an effect that may be due to slightly higher plasma triglyceride concentrations amongst the former. Postprandial lipemia is more profound and persistent when a saturated fat load replaces one of polyunsaturated fat [7]. Postprandial lipemia is also more profound and persistent when the fat load is consumed with alcohol [7,8]. Once again the enhancement of lipemia is more profound when polyunsaturated fat is replaced by saturated fat [7]. Comparison of the triglyceride fatty acid compositions of the fat load with those of the postprandial plasma showed them to be the same [9]. Thus, preprandial alcohol inhibits the lipolysis of the intestinally-derived lipoproteins, the chylomicrons. This is an interesting finding and when added to the observed effects of alcohol or acetate on adipose tissue lipolysis, indicates that alcohol ingestion inhibits both adipose tissue lipolysis and lipoprotein lipase (LPL). This is an unexpected finding given that adipose tissue lipolysis is inversely correlated with LPL activity; e.g., following a meal, increased LPL activity is associated with reduced adipose tissue lipolysis. On the basis of these observations, one can put forth the as yet untested hypothesis, “acetate inhibits LPL activity.” Given that household vinegar is dilute acetic acid, it is important to determine the effects of this common food ingredient on the metabolism of dietary fat.

### 2.3. Alcohol and High Density Lipoprotein Metabolism

Moderate alcohol consumption raises HDL-C concentrations [10]. A meta-analysis indicated that the consumption of 30 g (1 fluid oz.) of alcohol per day increases HDL cholesterol levels by a mean of 4 mg per deciliter, irrespective of the kind of alcohol consumed [11]. Persons who consume one to three drinks daily have higher HDL cholesterol levels and a lower risk of myocardial infarction than those who drink less, even after adjustment for other likely confounding factors [12]. The effects of alcohol consumption and exercise have been compared; both raise plasma HDL-C concentrations; interestingly, the effects of both alcohol consumption and exercise on plasma HDL-C concentrations were no different than the effects of each activity alone [9]. These findings suggest that mild-to-moderate alcohol consumption (≤one to two drinks per day) might be a reasonable consumption rate for some persons with low plasma HDL-C concentrations. However, the potential risks associated with this recommendation may outweigh the benefits in persons with hepatic dysfunction, a potential for addiction, psychosocial disorders, *etc*. Alternatives that raise plasma HDL-C in a cardioprotective way are needed. Currently there are no widely accepted and validated strategies for raising HDL-C in a cardioprotective manner.

## 3. G-Protein Coupled Receptor (GPCR) 43

The mechanism by which alcohol, via acetate, alters plasma lipids and lipoproteins is complicated because acetate is a precursor to many lipids such as sterols and glycerolipids, including triglycerides. Nevertheless, clues have emerged from studies of colonic fermentation, which produces short chained fatty acids that are ligands for several GPCRs. One of these, GPCR43, a receptor for acetate, is likely involved in immune and inflammatory responses because it is highly expressed in immune cells [13,14]. Importantly, and relevant to lipid metabolism, GPCR43 is also expressed in mouse adipocytes which, in response to acetate, exhibit reduced lipolysis, an effect that is not seen in adipocytes from GPCR43-null mice [15]. In addition, activation of GPCR43 by acetate reduces plasma free fatty acid levels in mice without the flushing effects seen by activation of GPCR109A with nicotinic acid [16]. Others showed that that GPCR43-deficient mice are obese on a normal diet, whereas mice overexpressing GPCR43 specifically in adipose tissue remain lean even when fed a high-fat diet [17]. Raised under germ-free conditions or after treatment with antibiotics, both types of mice have a normal phenotype. Moreover acetate activation of GPCR43 suppresses insulin signaling in adipocytes, which inhibits fat accumulation in adipose tissue and promotes metabolism of unincorporated lipids and glucose in other tissues. Thus, GPCR43 is a sensor for excess dietary energy, thereby controlling body energy utilization while maintaining metabolic homoeostasis and is a link between the metabolic activities of gut microbiota with host body energy homoeostasis. Thus, acetate via GPCR43 might regulate plasma lipid profiles and some components of metabolic syndrome (MetS).

### 3.1. Acetate as CVD Therapy

Plasma levels of acetate could increase up to 20-fold and into millimolar concentrations after alcohol ingestion [18,19]. Dietary acetic acid reduces serum cholesterol and triacylglycerols in rats fed a cholesterol-rich diet [20]. Ingestion of acetic acid as vinegar by rats lowers blood pressure, an effect that has not been tested in humans [21]. It is speculated that the risk of fatal ischemic heart disease in humans is lowered by the regular consumption of vinegar and oil salad dressings [21]. It is interesting to further speculate that perhaps the observed effects of ethanol on lipids, blood pressure, and CVD, of ethanol is, at least in part, mediated through its conversion to acetate and activation of GPCR43. Given the observed beneficial effects of raising HDL associated with alcohol consumption, the similar effects of nicotinic acid receptor and GPCR43 on adipocytes, and the proposed mechanism for HDL raising activities of nicotinic acid, the possible utility of GPCR43 as a potential target for the treatment of dyslipidemia should be further explored in the future. Tests in humans showing that oral acetate raises HDL-C might establish acetate as the HDL-C-raising effect of alcohol and suggest that acetate ingestion by humans is cardioprotective, an effect that may be due to inhibition of adipocyte lipolysis and suppression of MetS and to increased plasma HDL-C concentrations.

### 3.2. Antidiabetic Effects of Acetate

Before the introduction of modern hypoglycemic agents, diabetics used vinegar teas to control symptoms [21]. Several trials have revealed that the addition of small amounts of domestic vinegar (~25 g) to food, or vinegar taken with a meal reduce the glycemic index of carbohydrate food for people with and without diabetes [22,23,24]. This also has been expressed as lower glycemic index ratings in the region of 30% [25,26]. Vinegar (40 g) improves insulin sensitivity in normal and MetS subjects [27], an effect that would be expected to reduce net adipocyte lipolysis, thereby lowering plasma nonesterified fatty acid and triglyceride (TG) levels. Moderate daily alcohol intake is also associated with lower insulin secretion [28]. Thus, acetate from alcohol and in other food stuffs could be salutary and operate through its effects via adipose tissue-GPCR43. As potential anti-MetS, anti-diabetic and cardioprotective agents, acetate may offer other advantages over alcohol. Alcohol ingestion has also been linked to cancer of the upper gastrointestinal tract [29,30].

### 3.3. Alcohol and Pancreatitis

Acetate ingestion may not always be salutary. Hypertriglyceridemia (HTG) accounts for ~15% of pancreatitis cases in susceptible patients [31]; most other cases are idiopathic [32]. Currently, pancreatitis-susceptible patients with HTG are advised to avoid alcohol even though alcohol does not induce lipemia in HTG patients [33]; However, acetate, a component of prepared foods, could contribute to pancreatitis if, like alcohol, it enhances postprandial lipemia [3,7], which could trigger pancreatitis in unsuspecting HTG patients, who already exhibit impaired lipoprotein lipase activity. Acetate and its precursors in the food supply could underlie, in part, the idiopathic nature of pancreatitis, and therefore its identification as the culprit would equip physicians and susceptible patients with the information needed to avoid this debilitating and sometimes fatal disease.

### 3.4. Effects of Acute Alcohol on De Novo Lipogenesis (DNL) and Lipid Balances in Humans

Elegant quantitative studies of hepatic DNL and plasma acetate production on plasma lipids and whole body lipid balances in response to alcohol ingestion (24 g) by healthy men (*n* = 8) have been determined by stable isotope methods and indirect calorimetry. These studies yielded measures of DNL, lipolysis, conversion of alcohol to plasma acetate, and plasma acetate flux. Alcohol consumption increased the fractional contribution from DNL to VLDL-triacylglycerol palmitate from 2% to 30%, even though the absolute DNL rate represented <5% of the ingested alcohol. Most of the alcohol (~80%) cleared from plasma was converted to acetate entering plasma. Flux of acetate, which increased ~3-fold after alcohol consumption, likely triggered the decreased adipose tissue release of fatty acids into plasma 53% and reduced whole-body lipid oxidation by 73%. Thus, alcohol consumption modestly activates hepatic DNL, but hepatic acetate production and the release into plasma inhibits lipolysis and alters tissue fuel selection; acetate is the major quantitative fate of ingested ethanol. Although these studies show how alcohol modifies lipid metabolism, they remain confounded by the likelihood that the anti-diabetic and cardioprotective effects are mediated by acetate, effects that might by shrouded by separate effects of the precursors, alcohol and acetaldehyde. What is needed now is a similar stable isotope study in which labeled acetate replaces alcohol.

### 3.5. Mechanistic Model for the Salutary Effects of Acetate

**Figure 1:**
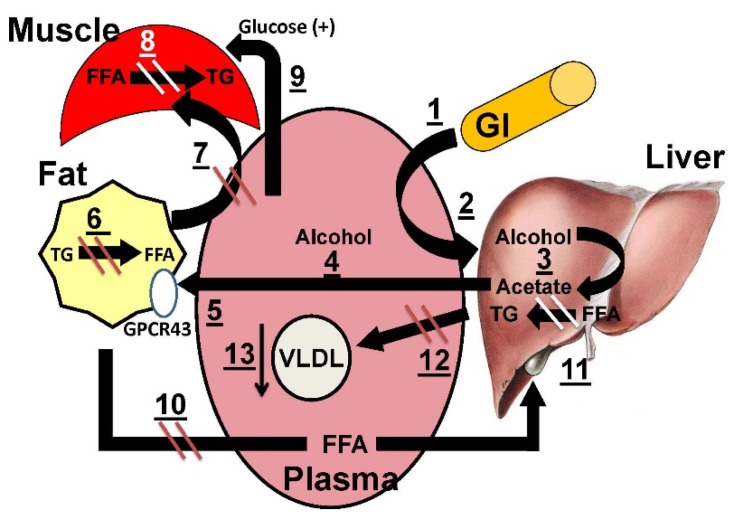
Hypothetical model for the effects of alcohol ingestion on plasma lipids and lipoproteins. See text for details.

Hypothetically, in the aforementioned context, acetate could induce multiple salutary effects in patients with MetS and/or insulin resistance. (Figure 1). Following its ingestion, alcohol transfers from the gastrointestinal (GI) tract to the plasma compartment (1), after which it enters the liver (2), where two successive dehydrogenase activities convert alcohol to acetaldehyde, and then to acetate (3). The liver releases acetate into the plasma (4), from whence it associates with adipocyte-GPCR43 (5), which initiates a series of signal transduction steps that inhibit lipolysis (6). With a decrease in lipolysis there is a subsequent reduction in fatty acid release into the plasma, which results in less FFA uptake by skeletal muscle (7), which reduces muscle lipid (8) and increases glucose disposal (9). The lower plasma FFA concentration (10) also reduces hepatic uptake, thereby reducing triglyceride (TG) synthesis (11) and VLDL-TG secretion (12); this produces a more hypotriglyceridemic state (13). There are other healthful downstream effects of reduced plasma TG. With lower VLDL-TG, there is less TG available for the exchange for LDL- and HDL-cholesteryl esters mediated by cholesteryl ester transfer protein. This also reduces the HDL- and LDL-TG so that they are no longer good substrates for hepatic lipase, which converts TG-rich LDL and HDL to their smaller, denser analogs. The hypothetical effects of alcohol ingestion on plasma lipids and lipoproteins are summarized in Table 1.

**Table 1:** Effects of Alcohol Ingestion on Lipids and their Metabolism.

Analyte	Effect
Plasma FFA	Decrease
Plasma alcohol	Increase
Plasma acetate	Increase
Plasma TG	Decrease
Plasma Glucose	Decrease
HDL-C	Increase
Small, dense LDL	Decrease

## 4. Conclusions

So, we return to our two opening questions. First, what are the mechanisms by which alcohol reduces the CVD rates? The answer is now clearer, but not totally clear. The reduction in CVD rates may have more to do with the insulinemic effects of acetate that are mediated by GPCR43, than with parallel changes in plasma HDL-C. Second, are there other healthful alternatives to alcohol consumption that could provide the positive CVD effects of alcohol without the occurrence of other diseases, including psychosocial effects that occur at high rates of consumption? Again, the answer cannot be stated with great confidence, but on the basis of the current data, one could hypothesize that acetate is the healthful alternative.

Despite much research on alcohol, its effects on human health are still not adequately understood. If the healthful effects of alcohol can be fully emulated by acetate, which does not have many of the unhealthy effects of alcohol, acetate ingestion might be the right medicine. Notably, larger doses of acetate *vs.* alcohol can be tolerated, so that healthful effects induced by alcohol might be increased by intake of a higher molar equivalent of acetate. However, more research on the effects of acetate on multiple metabolic pathways shown in Figure 1 and Table 1 need to be identified before such a plan could be implemented. Foremost among these would be to show that acetate alone increases plasma HDL-C concentrations and improves glucose disposal. In addition, stable isotope studies that uncover the underlying mechanisms should be conducted to guide the dose-optimization.
